# Molecular characterization of human adenovirus infection in Thailand, 2009–2012

**DOI:** 10.1186/1743-422X-10-193

**Published:** 2013-06-13

**Authors:** Punsinee Sriwanna, Thaweesak Chieochansin, Chanpim Vuthitanachot, Viboonsuk Vuthitanachot, Apiradee Theamboonlers, Yong Poovorawan

**Affiliations:** 1 Department of Pediatrics, Center of Excellence in Clinical Virology, Faculty of Medicine, Chulalongkorn University, Bangkok, 10330, Thailand; 2 Chum Phae Hospital, Chum Phae district, Khon Kaen province, 40130, Thailand

**Keywords:** HAdV, Hexon, Respiratory virus infection, Gastro viral infection, Epidemiology

## Abstract

**Background:**

Human adenovirus (HAdV) can cause a wide spectrum of human diseases worldwide.

**Methods:**

Using PCR and sequence analysis, we investigated HAdV infection prevalence in the Thai population for four years from January 2009 to December 2012. We collected Nasopharyngeal swab/aspirate (NP) specimens from patients in Bangkok, Khon Kaen, and Nakhon Si Thammarat province and fecal specimens only from Bangkok and Khon Kaen province.

**Results:**

We observed HAdV infection in 1.04% (82/7,921) of NP samples and in 5.84% (76/1,301) of fecal specimens. HAdV-B3 (32%) and HAdV-C1 (31%) were the genotypes most commonly associated with NP specimens followed by HAdV-C2 (13%) and HAdV-C5 (12%). In fecal specimens, we found that 25% harbored HAdV-F41 followed by HAdV-C1 (18%), HAdV-C2 (16%), and HAdV-B3 (13%). Out of all population subsets, children below the age of 3 years were the most likely to be HAdV positive (63.29%). In addition, HAdV infection occurred throughout the year without a seasonal distribution pattern, although HAdV infection of NP samples peaked from January-April while HAdV infection peaked from January to March and then again from May to July in fecal samples.

**Conclusions:**

This study has for the first time reported the HAdV infection rate in Thai NP and fecal specimens from 2009–2012. We observed that HAdV-B3 and HAdV-C1 were commonly found in NP specimens, and that HAdV-F41 was the most prevalence in fecal specimens in Thailand during the study period.

## Background

Human adenovirus (HAdV) was discovered in 1953 [1]. The virus can cause a wide spectrum of human diseases including respiratory tract infection, ocular infection, hemorrhagic cystitis, and gastroenteritis. HAdV infection is transmitted through inhalation, direct contact with small droplet aerosols or the fecal-oral route. HAdV infection occurs throughout the year. Worldwide, approximately 5-7% of respiratory tract infections (RTI) in children are ascribed to HAdV [2]. The clinical manifestations of HAdV infection commonly include fever, cough, nasal congestion and sore throat. In addition, with a prevalence ranging from 4-12%, this virus is the third most common cause of viral gastroenteritis in children, which causes diarrhea, vomiting and respiratory tract symptoms in half of the patients [3]. Although most infected patients are frequently asymptomatic or, the infection is mild and self-limited, HAdV infection can cause high mortality rates in both healthy individuals and immune-compromised patient [4].

HAdV is a non-enveloped virus composed of double stranded linear DNA. Virus particles range from 70–90 nm in size and belong to the family *Adenoviridae*, genus *Mastadenovirus*. Based on characteristics such as haemagglutination, length of the fiber gene, and GC content of its genome, 68 types of HAdV have been recently classified, which can be divided into seven different subgroups or species (A-G) [2,5-7].

Previous studies have reported that specific types are often associated with certain clinical symptoms, epidemiological settings and demographic risk groups. For example, HAdV-C1 – HAdV-B7 is commonly associated with RTI, while HAdVF40/41 is mostly found in gastroenteritis patients [3,8]. Morfin et al. showed that the antiviral drug response in-vitro varied according to genotype [9]. Thus, HAdV genotype diagnosis may be beneficial for prescribing more effective treatments in the future.

Although HAdV infection has been well researched in a number of countries across the world [10-18], knowledge on prevalence of HAdV infection in Thailand and South East Asia is limited. In Malaysia, an epidemiologic study showed that >2% of HAdV infections were found in patients’ NP and that the genotype was predominantly HAdV-C [12]. In addition, Shuvra and colleagues showed that the prevalence of HAdV between 2004 and 2005 was 1.9% in children with acute gastroenteritis in Bangladesh [19] while only one case was reported in Thailand in 2007 [20].

Therefore, the purpose of this study has been to molecularly characterize HAdV and determine its prevalence in Thailand between 2009 and 2012. Nasopharyngeal swab or aspirate and stool samples were collected from the patients with respiratory tract infection and acute diarrhea, respectively and PCR was used as the diagnostic tool. The resulting data will be useful to understand the molecular prevalence and seasonal distribution of HAdV infection in Thailand, and this information can be used to take preventive measures aimed at controlling future outbreaks.

## Results

### General findings

**Figure 1:**
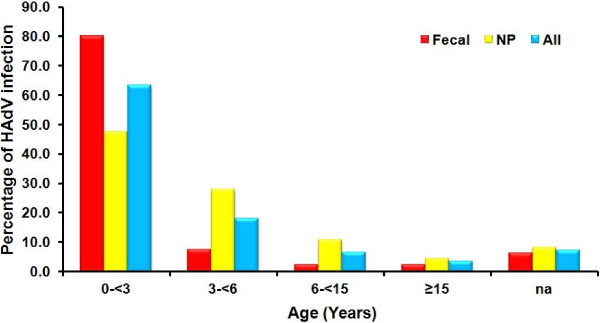
Age distribution of HAdV infection in Thailand.

**Figure 2:**
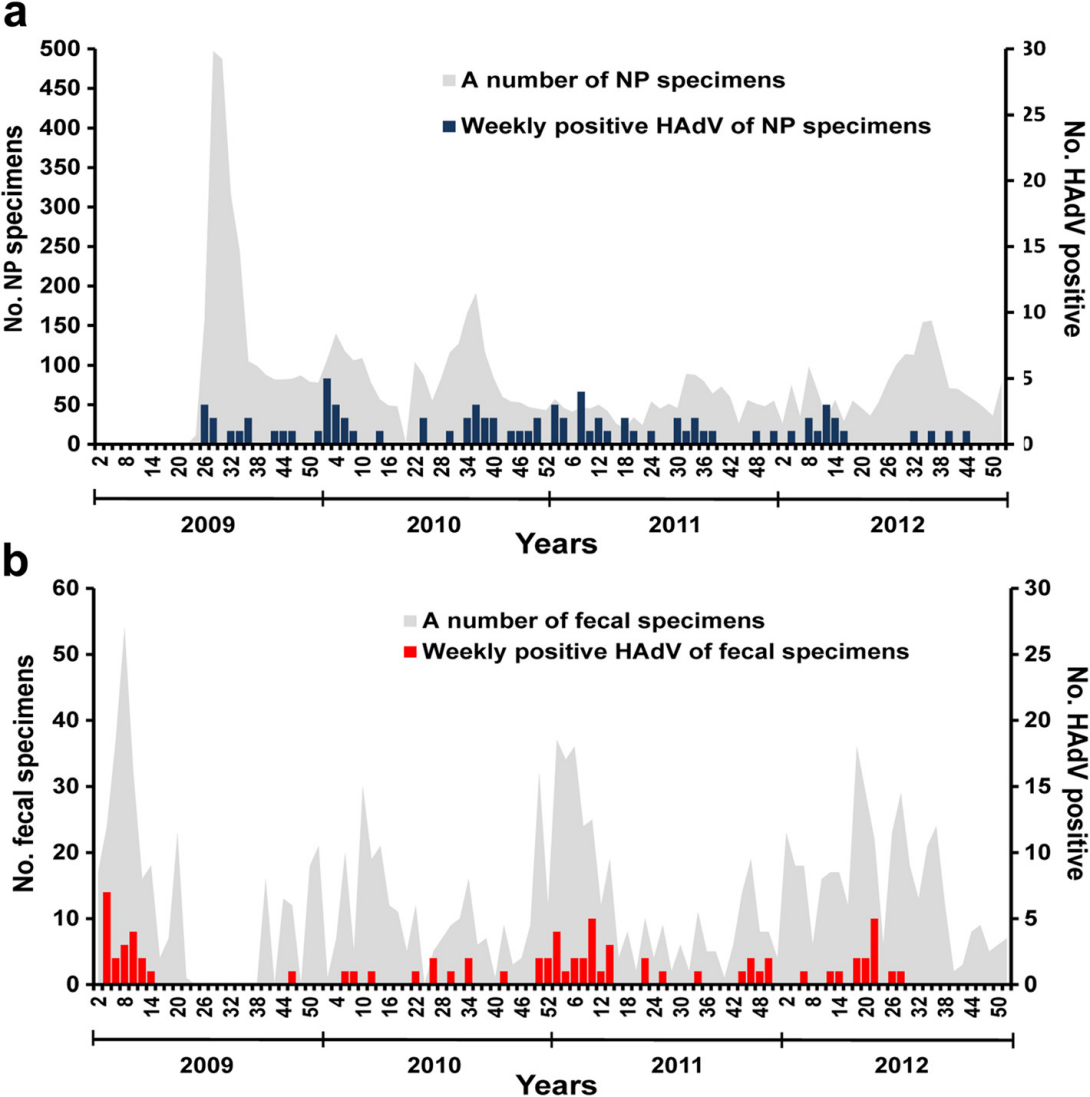
**Weekly HAdV positive. a**. Weekly HAdV-positive for nasopharyngeal specimens from patients, week 23, 2009 – week 52, 2012 (June 1, 2009-December 31, 2012). **b**. Weekly HAdV-positive for fecal specimens from patients, week 1, 2009 – week 52, 2012 (January 1, 2009-December 31, 2012).

Out of 7,921 NP specimens collected between August 2009 and December 2012, 82 (1.04%) samples were HAdV positive by PCR. Among the positive cases, 53.7% were male and 46.3% were female (1.16:1) (Tables 1 and 2). Children between 0 – < 3 years old represented the majority of infected patients at 47.6% (39/82) followed by children between the age of 3 – <6 years at 28.1% (23/82) and 6 – <15 years at 11% (9/82) (Figure 1). The mean age of HAdV infected patients was 4.86 years (min = 4 months, max = 43 years, SD =13.3, mode = 1, median = 2). The percentage of HAdV infection per year was; 0.52 (13/2497) in 2009, 1.29% (29/2246) in 2010, 1.97% (26/1318) in 2011 and 0.75% (14/1860) in 2012. HAdV infection occurred throughout the year with the peak period from January to March (winter – early summer) (Figure 2a).

**Table 1:** Demographic characteristics of participants

	**NP specimen (n = 7,921)**		**Fecal specimen (n = 1,301)**	
**Characteristic**	**No. specimens**	**Positive HAdV (%)**	**No. specimens**	**Positive HAdV (%)**
Sex				
Male, n (%)	4,103 (58.8)	44 (53.7)	776 (59.7)	49 (64.5)
Female, n (%)	3,818 (48.2)	38 (46.3)	525 (40.4)	27 (35.5)
Age(y)				
Median	5	2	1	1
Mode	1	1	1	1
Mean (SD)	9.76 (13.4)	4.86 (13.3)	3.14 (5.3)	2.93 (9.3)
Age group				
0- < 3, n (%)	2,194 (27.7)	39 (47.6)	1,019 (78.3)	61 (80.3)
3- < 6, n (%)	1,263 (15.9)	23 (28.1)	90 (6.9)	6 (7.9)
6- < 15, n (%)	1,909 (24.1)	9 (11.0)	29 (2.2)	2 (2.6)
≥15, n (%)	2,243 (28.3)	4 (4.9)	124 (9.5)	2 (2.6)
missing, n	312	7	39	5
Provinces				
Bangkok, n (%)	3,414 (43.1)	39 (1.1)	368 (28.3)	19 (5.2)
Khon Kaen, n (%)	3,414 (43.1)	31 (0.9)	933 (71.7)	57 (6.1)
Nakhon Si Thammarat, n (%)	1,093 (13.8)	12 (1.1)	-	-

**Table 2:** HAdV infection prevalence and distribution of age group

	**Total samples (n)**	**HAdV positive samples (n) (%)**
NP	7,921	82 (1.04)
Fecal	1,301	76 (5.84)
Age group		
0- < 3	3,213	100 (3.11)
3- < 6	1,353	29 (2.14)
6- < 15	1,938	11 (0.57)
≥15	2,367	6 (0.25)

From January 2009 to December 2012, HAdV was detected in 76/1,301 (5.84%) of fecal specimens with a male: female ratio of 1.82:1 (Tables 1 and 2). Children between 0 – < 3 years represented the majority of infected patients at 80.3% (61/76) followed by 3 – < 6 year olds and 6 – < 15 year olds at 7.9% (6/76) and 2.6% (2/76), respectively (Figure 1). The mean age of HAdV infected patients was 2.93 years (min = 26 days, max = 68 years, SD = 9.3, model = 1, median = 1). The HAdV positive rate per year was 6.39% (20/313) in 2009, 5.13% (14/273) in 2010, 8.89% (28/315) in 2011 and 3.50% (14/400) in 2012. HAdV infection was observed throughout the year with the peak period from January to March (winter – early summer) (Figure 2b).

### Molecular characterization and genotype of HAdV

**Figure 3:**
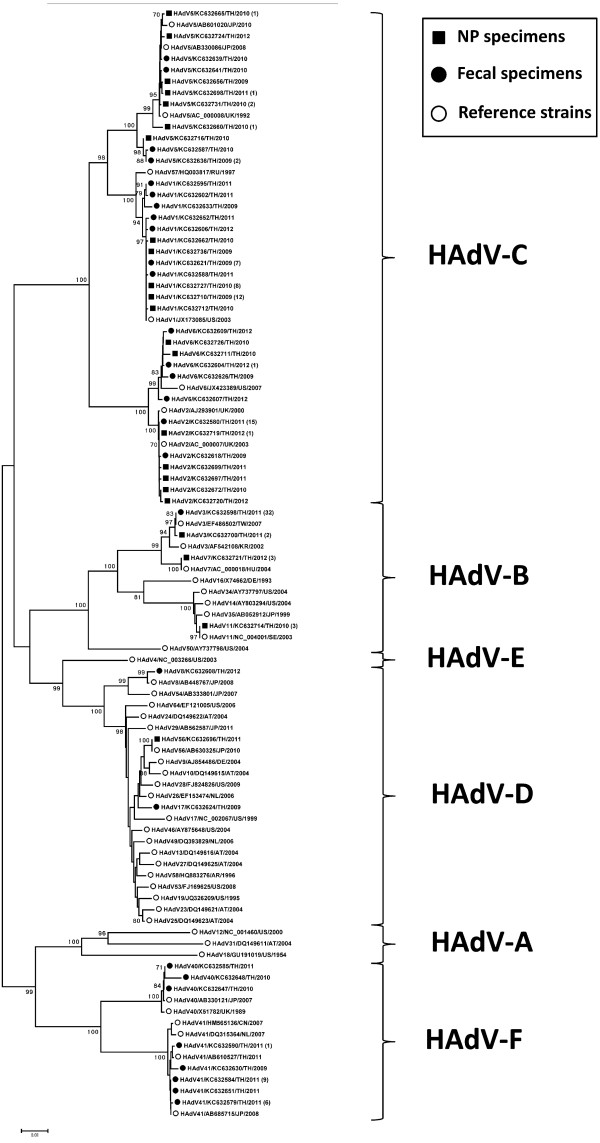
**Phylogenetic tree of HAdV in Thailand during 2009–2012.** Phylogenetic tree of adenovirus hexon gene sequences of clinical specimens and other reference strains. Boots-trap proportions (1,000 replications) are indicated as a percentage in each node. A number of identical nucleotide sequences were presented in parentheses.

**Figure 4:**
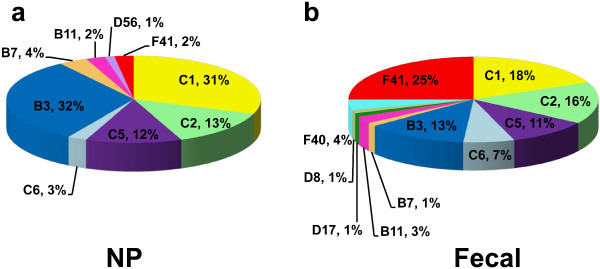
Distribution of HAdV genotypes among NP specimens (a), fecal specimens (b).

**Figure 5:**
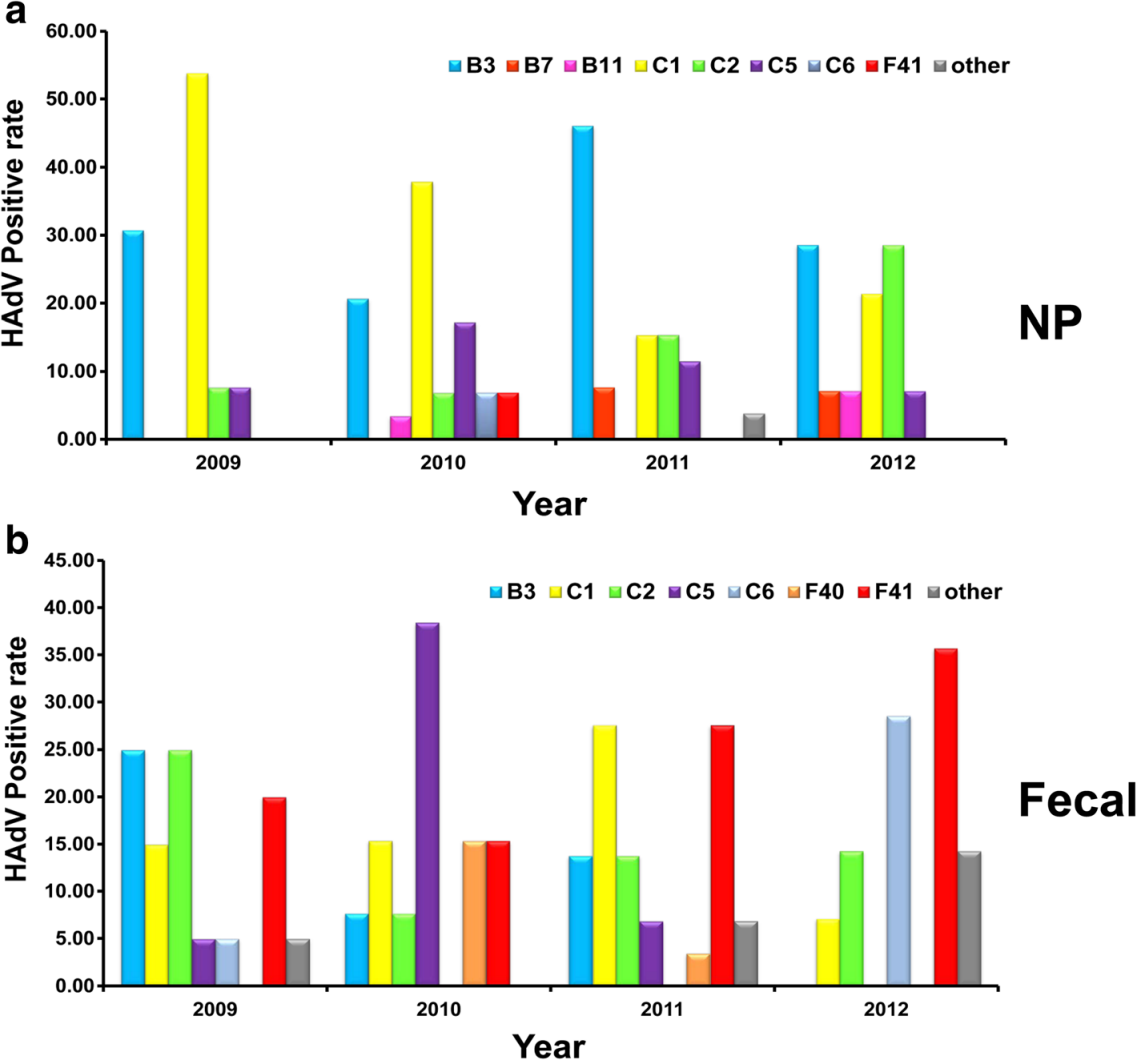
**Distribution of HAdV genotypes in NP specimens (a) and fecal specimens (b) during 2009 – 2012.** Each colour represents a separate genotype while the grey bar represents all others genotypes; HAdV-D56 in NP specimens and HAdV-B11, HAdV-C6, HAdV-D8, HAdV-D17 in fecal specimens.

A phylogenetic tree was constructed from the HAdV nucleotide sequences obtained in this study with the HAdV sequence stored in GenBank as the reference genome. This study showed that there were different patterns for each specimen (Figure 3). The most common HAdV genotypes found in NP specimens were HAdV-B3 32% (26/82), followed by HAdV-C1 31% (25/82), HAdV-C2 13% (11/82) and HAdV-C5 12% (10/82). Overall, HAdV subgroup C being the predominant subgroup found in NP specimens (Figure 4a). HAdV-B3 (35.90%) was the predominant genotype found in Bangkok followed by HAdV-C1 (28.21%), while in Khon Kaen province, HAdV-B3 (29.03%) and HAdV-C1 (29.03%) were the most common genotypes. We found the highest rate of HAdV-C1 infection (41.67%) followed by HAdV-B3 infection (25%) in Nakhon Si Thammarat. HAdV genotype prevalence fluctuated according to the study year. HAdV-B3, HAdV-C1, and HAdV-C2 were detected in NP throughout every year of the study (Table 3). In 2009, HAdV-C1 (53.85%) was most prevalent followed by HAdV-B3 (30.77%). In 2010, HAdV-C1 (37.93%) was predominant, followed by HAdV-B3 (20.69%) and HAdV-C5 (17.24%). In 2011, HAdV-B3’s prevalence was 46.15% followed by HAdV-C1 and HAdV-C2, which both had a prevalence of 15.38%. In 2012, HAdV-B3 (28.57%) and HAdV-C2 (28.57%) were most prevalent followed by HAdV-C1 (21.43%) (Figure 5a).

**Table 3:** **HAdV genotypes distribution in Thailand**, **2009**–**2012**

**HAdV genotypes**	**NP specimens (n)**	**Fecal specimens (n)**
B3	26	10
B7	0	1
B11	2	2
C1	25	14
C2	11	12
C5	10	8
C6	2	5
D8	0	1
D17	0	1
D56	1	0
F40	0	3
F41	2	19
Total	82	76

HAdV subgroup C was the most commonly observed genotype in fecal specimens at 51% (39/76). However, if each genotype is considered individually, HAdV-F41 was the most prevalent at 25% (19/76), followed by HAdV-C1 18% (14/76), HAdV-C2 16% (12/76) and HAdV-B3 13% (10/76). The least common genotypes were HAdV-B7, HAdV-D8 and HAdV-D17 at 1.3% (1/76) (Figure 4b). HAdV-F41 was most commonly found in Bangkok (26.32%) and Khon Kaen provinces (24.56%). The HAdV genotypes which we found to be present in fecal specimens in every year of the study were HAdV-F41, HAdV-C1 and HAdV-C2 by order of prevalence (Table 3). In 2009, the most prevalent genotypes were HAdV-B3 (25%), HAdV-C2 (25%) and HAdV-F41 (20%). In 2010, genotype HAdV-C5 (38.5%) had the highest prevalence. In 2011, HAdV-C1 (28.6%) and HAdV-F41 (28.6%) were the most common genotypes found, and in 2012, HAdV-F41 was found to have the highest prevalence (35.71%). Interestingly, while HAdV-C6 was detected in 5% of samples in 2009, it accounted for 28.57% of positive samples by 2012. On the other hand, HAdV-F40 was observed at a prevalence of 15.38% in 2010, decreased to an observed prevalence of 3.45% in 2011 and was not detected at all in 2012 (Figure 5b).

## Discussion

In this study, we investigated molecular character of HAdV infection in Thailand by PCR from January 2009 to December 2012. The 2 types of specimens used comprised 7,921 nasopharyngeal swab/aspirate (NP) samples collected from Bangkok, Khon Kaen, and Nakhon Si Thammarat province and 1,301 fecal specimens sent from Bangkok and Khon Kaen province. All of the specimens were collected anonymously through the influenza surveillance project and rotavirus diagnosis. Unfortunately, we could not clarify exactly which samples originated from outpatients or inpatients. However the majority of the samples were from outpatients. HAdV infection was detected in 1.04% of NP specimens. The rate of HAdV positive samples was similar to the level of infection in other Asian countries such as China 0.9% [10], and Malaysia 1.85% [12]. On the other hand, prevalence of HAdV infection in this study was lower than reported from non-Asian countries such as Brazil 7.1% [13], Peru 6.2% [14], Canada 7.73% [15], Columbia 5% [16]. In addition, Michal Mandelboim and colleagues found an infection prevalence of 18.3% in Israel; however, this study included immune deficient patients [17]. The male/female ratio (M/F) of HAdV infected NP samples in our study was 1.16:1,which is lower than previously reported from Israel 1.48:1 [17], Taiwan 1.3:1 [21], and Peru 1.3:1 [14]. The reason for these differences between the male to female ratio in different countries has not been identified. HAdV infection in this study mostly affected children under the age of 3, which is compatible with other studies of HAdV infection worldwide [12,15,17,19-25]. Interestingly, Children between the ages of 6 and 15 years old were infected with HAdV at a lower rate. In 2000, Cooper et al. showed that children had been infected early in life and therefore had acquired immunity to this infection [26]. During the four year period of our study, we observed that the highest rate of HAdV infection occurred in 2011, the same year HAdV outbreaks were reported in China [27] and Taiwan [21]. The prevalence of HAdV infection decreased in 2012. HAdV infection occurred throughout the year with no seasonal distribution pattern, which is similar to other reports [14,18,21,24,25]. HAdV infection of NP samples was detected from January-April (week 2–18, late winter – summer), which is similar to the results of other studies performed in Asia [11,19]. However, HAdV infection is most common in spring in Brazil [13], in fall and winter in Israel [17] and in winter in the US [23].

This study has been the first to investigate HAdV infection of NP samples from Thailand. HAdV subgroup C is the most common HAdV subgroup detected in our NP samples, which mirrors results from studies in Israel [17], Brazil [13], Peru [14] and Malaysia [12]. The second most common subgroup was HAdV subgroup B followed by HAdV subgroups F and D, respectively. However, in contrast to Thailand, HAdV subgroup B was predominant in USA, Japan, China, Korean, Taiwan and Canada [11,15,21,23,28-30]. The reason for this discrepancy in subgroup prevalence among nations has not yet been elucidated; however, it could be that HAdV-C is most common in tropical regions while subtropical and continental regions have a high frequency of HAdV-B. Unfortunately though, there is very little information concerning HAdV subgroup prevalence in other S.E. Asian countries besides Thailand. The study found that different genotypes of HAdV predominated in different provinces. The result showed that HAdV-B3 was the most prevalent genotype detected in Bangkok, whereas, HAdV-B3 and HAdV-C1 were found to be co-predominant in Khon Kaen, while, HAdV-C1 was commonly found in Nakhon Si Thammarat. These results are similar to a study carried out by Qurei and Mandelboim who reported that the predominant genotypes in Palestine and Israel were HAdV-B3 and HAdV-C1, respectively [17,31]. Overall, HAdV-B3 and HAdV-C1 are the most common genotypes followed by HAdV-C2 and HAdV-C5 while it was rare to find subgroups HAdV-B7, HAdV-C6, HAdV-B11, HAdV-F41 in NP specimens. Averaging over the four year period of our study, we found that HAdV-B3 and HAdV-C1 had equal infection rates, which has not been reported before. HAdV-B3 is the predominant genotype prevalent in USA [23], China [11,27], and Canada [15] while genotype HAdV-C1 is the predominant genotype in Malaysia [12] and Israel [17], although HAdV genotype distribution can change on a yearly basis. Infection of HAdV-B3, HAdV-C1, HAdV-C2, and HAdV-C5 were mainly found in NP specimens throughout the duration of our study which is similar to results from earlier studies of HAdV infection in other regions [11-15,17,21]. In 2009, HAdV-C1 accounted for more than 50% of infections followed by HAdV-B3. In contrast, the study of Tsung-Pei Tsou in the same year found that HAdV-B3 was the most common genotype followed by HAdV-C1 [21]. In 2010, prevalence of HAdV-C1 was slightly decreased but its prevalence was still higher than the prevalence of HAdV-B3 and HAdV-C5. In 2011, HAdV-B3 was predominant. This data was associated with an outbreak in Taiwan (73%) and China [21,27]. In 2012, percentage of HAdV-B3 infection dropped by half from that of the previous year and the rate of HAdV-C2 infection increased two fold compared to the year before.

The HAdV infection rate of fecal samples was 5.84%, which is a four-fold increase in comparison with the results of the previous study in Thailand in 2007 (1.5%), although that study had been based on a smaller population size and study period than the present one [18]. However, the percentage of HAdV infection in this study is similar to other countries, for example, Korea 2.6%, and Japan 4.8% [24,25]. The M/F ratio for HAdV infection of fecal samples was 1.82:1, which is an increase in comparison with the 2007 Thailand study (1:1), but similar to the previous report of Japan and Canada [15,25]. The incidence of HAdV infection was highest in children under 3 years of age. HAdV infection of fecal samples peaked from January to March and then again from May to July, similar to past reports in Thailand [20], Japan [32], and Bangladesh [19]. Ji W and colleagues have reported that the seasonal distribution pattern of respiratory viruses depends on the respective country’s weather patterns [33].

HAdV subgroup C was the predominant genotype found in infected fecal and NP samples followed by HAdV subgroups F, B, and D. This data diverges from Kittigul et al’s study which found HAdV-F to be the predominant subgroup [20]. In contrast, we found that HAdV-F41 was the most common HADV type in Bangkok and Khon Kaen and accounted for 25% of infected samples, which is similar to results from other countries such as Korean [24], Japan [32]. This subgroup has also been associated with acute diarrhea in infants and children [34]. On the other hand, a report from Bangladesh in 2004–2005 by Dey SK and colleagues stated that HAdV-F40 was the predominant subgroup found, although they did not observe HAdV-F41 [19]. In our study, we also found HAdV genotypes HAdV-C1, HAdV-C2, HAdV-B3, HAdV-C5, HAdV-C6, HAdV-F40, HAdV-B11, HAdV-B7, HAdV-D8 and HAdV-D17 in order of decreasing prevalence. This is in contrast to Kittigul’s study, which only observed genotypes HAdV-F41, HAdV-C2 and HAdV-D38. However, this may be due to the fact that our study lasted for a longer period and analyzed more samples. HAdV-F41, HAdV-C1 and HAdV-C2 were mostly observed in fecal samples. In 2009, HAdV-B3, HAdV-C2 and HAdV-F41 showed the highest frequency of infection. In 2010, HAdV-C5 was the predominant genotype after a 6-fold increase in prevalence from the previous year. HAdV-C1 and HAdV-F41 were the most common genotypes in 2011, although HAdV-C6 had not been found two years ago. Although HAdV-F40 is an enteric adenovirus like HAdV-F41, we found it to be less common than HAdV-F41. We found HAdV-F40 in 2010 and 2011 but not in 2012. Fecal specimens have been distinctively infected by non-enteric adenovirus because HAdV can be transmitted by the fecal oral route and the HAdV-F41 infection rate was most common as in previous study [35].

## Conclusions

In conclusion, because the information concerning HAdV infection in Thailand and the Southeast Asia region is insufficient, this study has for the first time reported the HAdV infection rate in Thai NP and fecal specimens from 2009–2012. Furthermore, we analyzed the age distribution, seasonal pattern, HAdV genotype and annual distribution of HAdV. HAdV-B3 and HAdV-C1 were commonly found in the NP specimens, and that HAdV-F41 was the most prevalence in the fecal specimens. We recommend surveillance and prevention of HAdV infection in early childhood. In the future, the study period of HAdV in this region should be increased in order to provide more epidemiologic information for therapeutic approaches and vaccine development.

## Methods

### Study population

**Figure 6:**
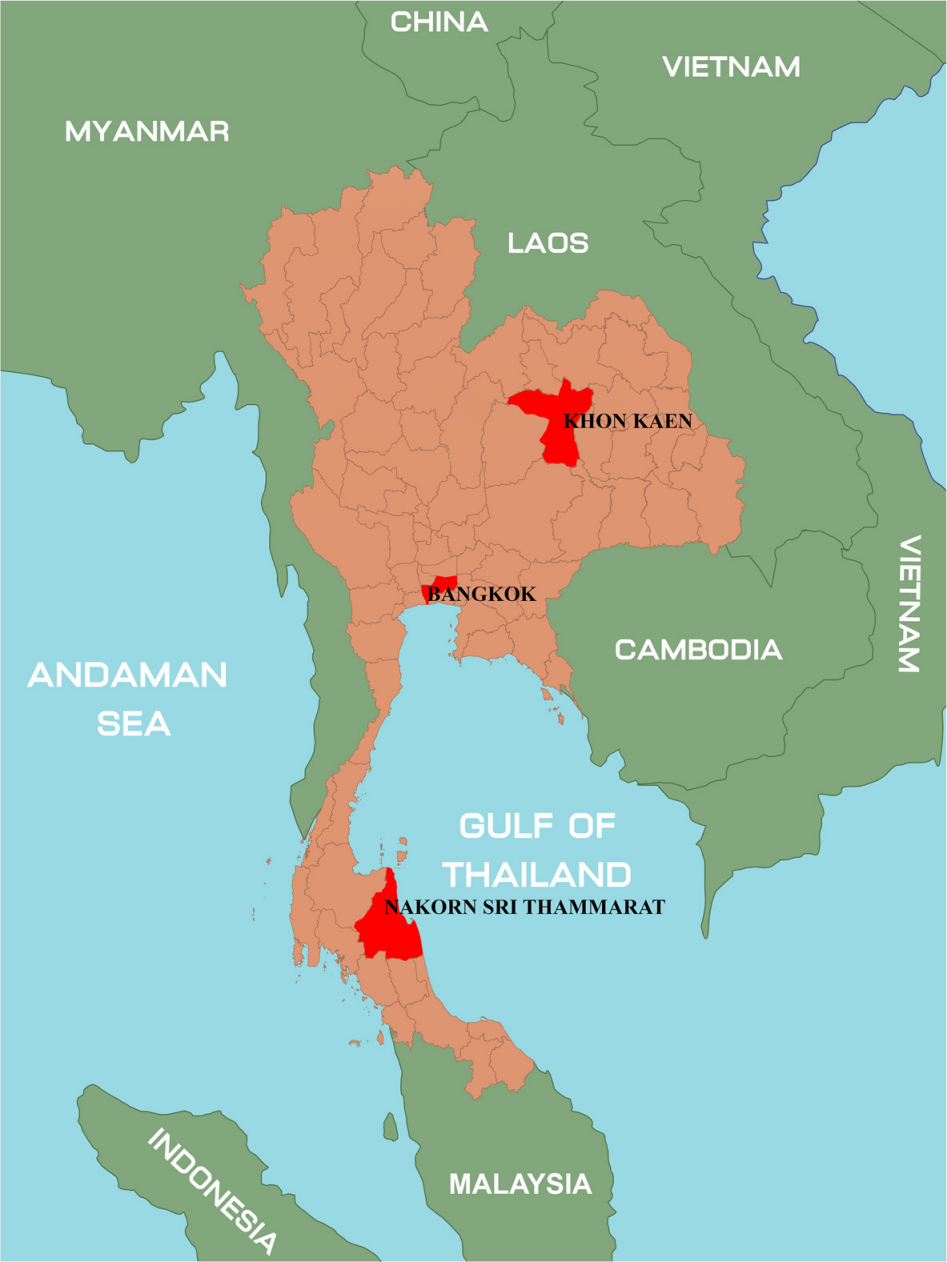
**A map of Thailand.** The detailed map of Thailand show provinces of Bangkok, Khon Kaen and Nakhon Si Thammarat, where the specimens were sent to Center of Excellence in Clinical Virology.

A total of 7,921 respiratory samples were collected as part of the routine influenza surveillance of both inpatients and outpatients with influenza-like illness (fever more than 38°C accompanied by respiratory symptom such as runny nose, sore throat, and cough). The patients’ age ranged from 1 month to 93 years old. Respiratory samples included nasopharyngeal (NP) aspirates (501 samples) and NP swabs (7,240 samples). The period of sample collection was from August 2009 to December 2012. These respiratory samples were gathered from three different places in Thailand; 3,414 samples from Bangkok province, 3,414 samples from Khon Kaen province and 1,093 samples from Nakhon Si Thammarat province (Figure 6). NP aspirates or swabs were placed into viral transport media (VTM) at the site of collection and then sent to the Center of Excellence in Clinical Virology, Department of Pediatrics, Faculty of Medicines, Chulalongkron Univesity. All samples were collected immediately upon arrival, and stored at −70°C until further use.

1,301 fecal samples were collected from inpatients with acute diarrhea as part of the routine rotavirus surveillance protocol. The patients’ age ranged from 26 days to 97 years. Samples were collected between January 2009 and December 2012 from Bangkok and Khon Kaen province. Fecal specimens were processed immediately upon arrival at the laboratory. Briefly, stool samples were diluted with PBS (1:10) and centrifuged at 4,000 rpm for 10 minutes. Subsequently, the supernatant was collected and stored at −70°C until further use.

## Human adenovirus detection

### DNA extraction

Viral DNA was extracted using the Viral Nucleic Acid Extraction Kit (RBC Bioscience, Taiwan). According to the manufacturer’s instruction, 200 μl of each sample was used, and 50 μl of nucleic acid free water was used to elute the virus genome in the final step. The DNA was then stored at −20°C until further use.

### HAdV detection

HAdV was detected by semi-nested PCR performed on *hexon* gene (modified from Xu et al.) [36]. ADV_FO (5′AYG CYA MCT TYT TYC CCA TGG C 3′) and ADV_R (5′AAR CCC TGR TAN CCD ATR TTG TA 3′) primers were used as the first round PCR primers. Two microliters of DNA were added into each PCR mixture which consisted of 1X Perfect TaqMasterMix (5 PRIME, Darmstadt, Germany), 10 mM of forward and reverse primers, and distilled water to a final volume of 25 μl. The PCR program used was as follows: 94°C for 3 min, then 40 cycles with 94°C for 18 s, 53°C for 21 s and 72°C for 1.30 min with a final extension at 72°C for 10 min. Then 1 μl of first round PCR product was subjected to second round PCR with the only differences being the forward primer used (5′TYT TYC CCA TGG CNC ACA ACA C 3′) and an annealing step at 55°C. The PCR products were subjected to 2% agarose gel electrophoresis and then stained with ethidium bromide solution. The stained gel was visualized under an UV-transilluminator with the expected band visible at 482 bp.

### HAdV sequencing and characterization

For characterization, positive HAdV samples had their *hexon* gene amplified. The method was modified from Lu X and Erdman’s study of 2006 [37]. We used the following primers: ADV_F2 (5′ TTY CCC ATG GCN CAC AAC AC 3′) and ADV_R2 (5′ GYY TCR ATG AYG CCG CGG TG 3′). Two microliters of DNA were added to a PCR mixture consisting of 1X Perfect TaqMasterMix (5 PRIME, Darmstadt, Germany), 10 mM of forward and reverse primers, and distilled water to a final volume of 25 μl. The PCR program was as follows: 94°C for 3 min followed by 40 cycles of 94°C for 30 s, 50°C for 30 s, and 72°C for 1.45 min with a final extension at 72°C for 10 min. PCR products (956 bp) were then purified, separated by 2% agarose gel electrophoresis and excised using the HiYieldTM Gel/PCR DNA fragment extraction kit (RBC Bioscience, Taiwan). Samples were then sent to First Base laboratories SDNBHD (Selangor Darul Ehsan, Malaysia) for sequencing. The nucleotide sequences from this study were submitted to the GenBank database under accession numbers KC632579-KC632736.

### Data and sequence analysis

The HAdV nucleotide sequences were analyzed using Chromas lite version 2.01, BioEdit version 7.0.4.1, NCBI BLAST software (http://blast.ncbi.nlm.nih.gov/) and Clustal X version 1.83. MEGA 5.05 software was used for phylogenetic analysis of aligned sequences. The phylogenetic tree was generated using the Neighbor-Joining (NJ) algorithm. The credibility of the phylogenetic tree was tested by applying a bootstrap test with 1,000 replications.

### Ethical considerations

All stored samples in this retrospective study were anonymous and acquired with permission from the Director of Chulalongkorn King Memorial hospital. The study protocol was approved by the Institutional Review Board, Faculty of Medicine, Chulalongkorn University, Thailand. (IRB No. 281/55) and the need for consent was waived because the samples were anonymous.

## Competing interests

The authors declare that they have no competing interests.

## Authors’ contributions

PS performed the experiment. CV and VV collected samples. YP, PS, TC and AT designed the study and conducted analysis and interpretation of the data. YP, PS, TC and AT revised the manuscript. All authors read and approved the final manuscript.
